# THE MEDIATING EFFECTS OF LIFESTYLES AND HEALTH CAPABILITIES ON THE RELATIONSHIP BETWEEN AGING AND LIFE SATISFACTION

**DOI:** 10.1093/geroni/igz038.3141

**Published:** 2019-11-08

**Authors:** Dongwook Cho

**Affiliations:** Alcorn State University, Lorman, Mississippi, United States

## Abstract

The importance of life satisfaction has been getting significant attention as the older population has increased rapidly. There are many studies to examine the relationship between older adults’ past and current lifestyles and life satisfaction levels that resulted to be either positively or negatively associated with. It is also well documented in the literature that aging is negatively associated with life satisfaction level due to the decrease in health capabilities. However, little study has been researched the direct and indirect relationships among advancing age, lifestyles, health capabilities, and life satisfaction levels. The purpose of the study was to examine the mediating effects of lifestyles and health capabilities on the relationship between aging and older adults’ life satisfaction levels. A total of 290 older adults completed the self-administered questionnaire of lifestyles and life satisfaction levels and their health capability assessments were evaluated at three clinical research centers in the U.S. Multiple regression test was utilized to analyze the data. The results showed the direct effects of advancing age [B =1.196, t=2.608, p=.010] and engagement in exercise or physical activity [B=-2.684, t=-3.071, p=.002] on life satisfaction levels among older adults. However, there were no mediating effects of lifestyles and health assessments on the relationship between age difference and life satisfaction levels among older adults. The results suggest the advancing age itself would be the strongest factor in older adults’ life satisfaction. Additionally, the findings also suggest that exercise and physical activity can enhance older adults’ life satisfaction levels as a supplemental factor.

